# Genome-wide analysis and expression profiling of the HD-ZIP gene family in kiwifruit

**DOI:** 10.1186/s12864-024-10025-7

**Published:** 2024-04-09

**Authors:** Kai-yu Ye, Jie-wei Li, Fa-ming Wang, Jian-you Gao, Cui-xia Liu, Hong-juan Gong, Bei-bei Qi, Ping-ping Liu, Qiao-sheng Jiang, Jian-min Tang, Quan-hui Mo

**Affiliations:** Guangxi Key Laboratory of Functional Phytochemicals Research and Utilization, Guangxi Zhuang Autonomous Region and Chinese Academy of Sciences, Guangxi Institute of Botany, Guilin, 541006 China

**Keywords:** Kiwifruit, HD-Zip, Gene family, Kiwifruit bacterial canker disease, Postharvest

## Abstract

The homeodomain-leucine zipper (HD-Zip) gene family plays a pivotal role in plant development and stress responses. Nevertheless, a comprehensive characterization of the HD-Zip gene family in kiwifruit has been lacking. In this study, we have systematically identified 70 HD-Zip genes in the Actinidia chinensis (Ac) genome and 55 in the Actinidia eriantha (Ae) genome. These genes have been categorized into four subfamilies (HD-Zip I, II, III, and IV) through rigorous phylogenetic analysis. Analysis of synteny patterns and selection pressures has provided insights into how whole-genome duplication (WGD) or segmental may have contributed to the divergence in gene numbers between these two kiwifruit species, with duplicated gene pairs undergoing purifying selection. Furthermore, our study has unveiled tissue-specific expression patterns among kiwifruit HD-Zip genes, with some genes identified as key regulators of kiwifruit responses to bacterial canker disease and postharvest processes. These findings not only offer valuable insights into the evolutionary and functional characteristics of kiwifruit HD-Zips but also shed light on their potential roles in plant growth and development.

**Supplementary Information:**

The online version contains supplementary material available at 10.1186/s12864-024-10025-7.

## Introduction

Transcription factors (TFs) that contain conserved DNA binding domains play a critical role in regulating the expression patterns of target genes by binding to specific cis-elements in the promoter regions of target genes, thereby affecting tissue development and cell differentiation in eukaryotic organisms [1]. The homeodomain-leucine zipper (HD-Zip) gene family is a plant-specific class of TFs that have been identified in various plant species, including *Arabidopsis thaliana* [2, 3], rice (*Oryza sativus*) [4], tomato (*Solanum lycopersicum*) [5], Brassica rapa [6], maize (Zea mays) [7]. The HD-Zip family comprises two conserved domains, the homeodomain and leucine zipper domain [8]. The homeodomain (HD) consists of 60 amino acid residues with highly conserved sequences that bind to the target DNA [9], while the leucine zipper (LZ) domain comprises 35–42 amino acid residues and influences the formation of protein dimers [10]. Utilizing the sequence characteristics of HD-Zip proteins, the HD-Zip family can be classified into four distinct subfamilies, namely HD-Zip I to IV [8–10]. In addition to the HD and LZ domains, HD-Zip proteins belonging to different subfamilies also possess other conserved domains, resulting in functional diversification of HD-Zip proteins [2–4].

The HD-Zip I subfamily primarily regulates plant leaf development and responses to external stimuli, such as temperature fluctuations, drought, and osmotic pressure [9, 11]. Four HD-Zip I genes (AtHB6/7/12/13) in Arabidopsis have been shown to regulate plant responses to drought and abscisic acid (ABA) treatment [12–15]. AtHB52 also affects plant responses to light and photomorphogenesis [3], while AtHB1 is regulated by short-day photoperiods and promotes hypocotyl and root elongation [16]. Furthermore, AtHB21/40/53 have been found to negatively regulate bud formation [17].

HD-Zip proteins belonging to the HD-Zip II subfamily contain the CPSCE (Cys-Pro-Ser-Cys-Glu) domain and a conserved N-terminal [8]. The HD-Zip II subfamily mainly regulates plant responses to light and auxin stimuli [8]. AtHB4 and HAT3 can be induced by auxin and are involved in shade-induced growth in *Arabidopsis* [18]. Previous studies have shown that the AtHAT3, AtHB2, and AtHB4 genes co-regulate shoot apical meristem (SAM) formation and cotyledon development in *Arabidopsis* seedlings [19]. The *athb4/hat3* double mutant produces severely abaxialized leaves [18].

The HD-Zip III subfamily is characterized by the presence of the steroidogenic acute regulatory protein-related lipid transfer (START) domain, which contains the SAD (START-adjacent domain) and MEKHLA (Met-Glu-Lys-His-Leu-Ala) motifs [8, 20]. This subfamily mainly plays a role in meristematic formation, lateral organogenesis, polar auxin transport, and vascular system development [20]. In Arabidopsis, the HD-Zip III subfamily includes five HD-Zip genes (*REV*, *PHB*, *PHV*, *AtHB15*, and *AtHB8*), and all five genes have been shown to directly regulate vascular development [20]. Additionally, three genes (*REV*, *PHB*, and *PHV*) also contribute to controlling the abaxial-adaxial patterning of lateral organs [21].

HD-Zip proteins belonging to the HD-Zip IV subfamily contain only the SAD motif and are primarily involved in regulating anthocyanin accumulation, cell differentiation, root development, and trichome formation [8–10, 18]. Previous studies have shown that *AtML1* and *AtPDF2* play a role in regulating shoot epidermal cell differentiation in Arabidopsis [8, 10]. Meanwhile, *AtGL2* and *AtHB10* negatively regulate hair formation to determine trichome and root-hair distribution patterns [22].

The *Actinidia* genus comprises 54 species and 75 taxa, and is particularly well-known for its most famous fruit, kiwifruit [23]. Kiwifruit has become a popular fruit worldwide due to its high vitamin C content and abundant minerals [24, 25]. Recently, whole-genome de novo sequencing projects and transcriptome sequencing data of *A. chinensis* (Ac) and *A. eriantha* (Ae) have been completed, revealing significant variation in flowering time and other vital traits between the two species [26–28]. The HD-Zip gene family has been shown to play important roles in plant development and stress responses [8–10]. However, no systematic investigation or functional analysis of the HD-Zip gene family has been reported in kiwifruit. Therefore, in this study, we comprehensively identified the HD-Zip gene family in the genomes of *A. chinensis* and *A. eriantha*, and systematically analyzed their gene structures, motif compositions, and chromosomal distributions in both kiwifruit species.

In this study, we aimed to comprehensively analyze the HD-Zip gene family in the *A. chinensis* and *A. eriantha* genomes. Our study investigated the gene structure, motif compositions, and chromosomal distributions of the HD-Zip gene family for both species. We also studied the phylogenetic relationships and evolution patterns of the HD-Zip gene family in these two kiwifruit species. In addition, we conducted cis-elements analysis and examined the expression patterns of the HD-Zip genes in various tissues and under different stress conditions. Our findings provide valuable insights into the potential functions of the HD-Zip genes in these two kiwifruit species.

## Results

### Identification and characterization of HD-Zip proteins in kiwifruit

**Fig. 1:**
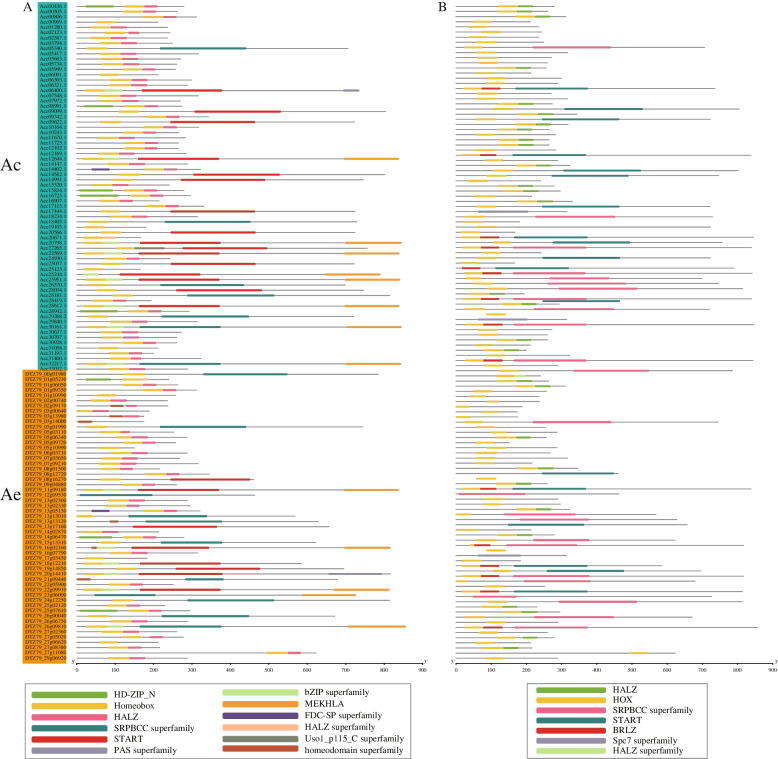
Conserved domains of HD-Zip genes in kiwifruit predicted by CDD **A** and SMART **B** Ac, *A. chinensis*; Ae, *A. eriantha*

To identify HD-Zip proteins in kiwifruit, we used the HMMER 3.0 software to search for HD-Zip proteins from the Ac and Ae genomes based on the HD-domain profile (PF00046) and LZ domain profile (PF02183) [27]. We found a total of 70 and 55 putative HD-Zips in Ac and Ae, respectively (Fig. 1 and Table S1). We confirmed that all putative HD-Zips in Ac and Ae contained the homeodomain and LZ domain Pfam and CD-search, as well as other conserved domains such as SRPBCC, START, and MEKHLA (Fig. 1). The coding sequence (CDS) length of *AcHB* genes ranged from 498 to 2535 bp, and the corresponding length of *AeHB* genes varied from 453 to 2568 bp (Table S1). The predicted length of AcHB proteins ranged from 166 to 845 amino acids, and the corresponding length of AeHB proteins ranged from 151 to 856 amino acids (Tables 1 and 2). The molecular weight range of AcHB proteins was from 19,322.87 to 93,075.14 Da, and the range for AeHB proteins was from 17,726.03 to 93,730.17 Da (Tables 1 and 2). The theoretical isoelectric point (pI) for AcHB proteins varied from 4.60 to 9.16, and the range for AeHB proteins was from 4.61 to 9.43 (Tables 1 and 2). Most AcHB proteins (63 out of 70) and AeHB proteins (44 out of 55) were predicted to be located in the nucleus (Tables 1 and 2).

**Table 1:** Protein composition and physiochemical characteristics of HD-Zip proteins in Ac

Name	Genome id	Protein Length (aa)	MW (Da)	pI	GRAVY	Predicted Localiaztion	Subfamily
AcHB1	Acc00436	279	30772.22	8.14	-0.823	Nuclear	II
AcHB2	Acc00505	260	29032.15	8.07	-0.837	Nuclear	II
AcHB3	Acc00806	312	35127.14	8.03	-0.786	Nuclear	II
AcHB4	Acc00969	211	24604.56	5.76	-0.956	Nuclear	I
AcHB5	Acc01280	236	26846.54	5.45	-0.950	Nuclear	I
AcHB6	Acc02123	243	27716.88	8.91	-0.841	Nuclear	I
AcHB7	Acc02587	236	27155.85	5.70	-1.118	Nuclear	I
AcHB8	Acc03794	249	28332.29	5.31	-0.745	Nuclear	I
AcHB9	Acc05340	706	78820.75	6.12	-0.360	Nuclear	IV
AcHB10	Acc05417	317	35706.89	4.82	-0.775	Nuclear	I
AcHB11	Acc05683	271	30919.99	4.60	-0.801	Nuclear	I
AcHB12	Acc05734	260	29657.94	5.69	-0.859	Nuclear	I
AcHB13	Acc05949	257	29090.36	6.85	-0.824	Nuclear	II
AcHB14	Acc06091	212	24470.45	6.05	-0.896	Nuclear	I
AcHB15	Acc06303	299	34522.07	4.81	-0.886	Nuclear	I
AcHB16	Acc06321	289	33201.67	5.88	-0.935	Nuclear	I
AcHB17	Acc06400	735	80655.73	6.25	-0.251	Nuclear	III
AcHB18	Acc07548	270	30466.51	4.80	-0.757	Nuclear	I
AcHB19	Acc07972	317	35647.71	4.83	-0.803	Nuclear	I
AcHB20	Acc08991	274	30430.83	7.58	-0.786	Nuclear	II
AcHB21	Acc09099	804	87389.54	5.97	-0.228	Nuclear	IV
AcHB22	Acc09342	343	37348.07	8.49	-0.837	Nuclear	II
AcHB23	Acc09622	723	79599.46	5.71	-0.331	Nuclear	IV
AcHB24	Acc10164	318	35799.10	8.03	-0.697	Nuclear	II
AcHB25	Acc10243	265	29557.75	7.54	-0.852	Nuclear	II
AcHB26	Acc11670	283	32991.53	5.66	-0.900	Nuclear	I
AcHB27	Acc11725	265	29593.03	7.58	-0.670	Nuclear	II
AcHB28	Acc12102	265	29515.07	8.39	-0.606	Nuclear	II
AcHB29	Acc12169	284	32683.15	5.32	-0.895	Nuclear	I
AcHB30	Acc12644	838	92141.61	5.97	-0.119	Plasma Membrane	III
AcHB31	Acc14147	289	33033.61	6.27	-0.855	Nuclear	I
AcHB32	Acc14402	322	36010.98	6.56	-0.770	Nuclear	II
AcHB33	Acc14582	801	87163.10	5.97	-0.264	Nuclear	IV
AcHB34	Acc14991	746	82410.66	5.79	-0.304	Nuclear	IV
AcHB35	Acc15520	241	27691.91	8.70	-0.813	Nuclear	I
AcHB36	Acc15834	279	31266.65	8.20	-0.797	Nuclear	II
AcHB37	Acc16725	296	33395.19	8.42	-0.895	Nuclear	II
AcHB38	Acc16907	215	24385.12	8.71	-0.806	Nuclear	I
AcHB39	Acc17115	331	36249.90	9.02	-0.811	Nuclear	II
AcHB40	Acc17444	723	79635.47	5.57	-0.327	Nuclear	IV
AcHB41	Acc18234	314	35679.85	4.61	-0.909	Nuclear	I
AcHB42	Acc18405	729	79967.89	6.07	-0.278	Nuclear	IV
AcHB43	Acc19105	181	20758.27	8.99	-0.783	Nuclear	I
AcHB44	Acc20566	723	79280.91	5.66	-0.329	Nuclear	IV
AcHB45	Acc20671	167	19709.02	7.72	-0.883	Nuclear	I
AcHB46	Acc20798	845	93075.14	5.93	-0.149	Nuclear	III
AcHB47	Acc22265	756	83707.70	5.99	-0.449	Nuclear	IV
AcHB48	Acc22569	839	92167.50	5.72	-0.077	Plasma Membrane	III
AcHB49	Acc24930	242	27780.92	6.54	-0.879	Nuclear	I
AcHB50	Acc25037	722	79259.02	5.64	-0.303	Nuclear	IV
AcHB51	Acc25123	166	19322.87	8.19	-0.747	Nuclear	I
AcHB52	Acc25244	790	86661.89	5.75	-0.077	Plasma Membrane	III
AcHB53	Acc25951	841	92148.16	5.93	-0.079	Plasma Membrane	III
AcHB54	Acc26570	699	78775.58	8.32	-0.263	Nuclear	IV
AcHB55	Acc28034	746	82596.76	6.06	-0.235	Nuclear	IV
AcHB56	Acc28181	814	90265.48	5.35	-0.360	Nuclear	IV
AcHB57	Acc28419	194	22091.10	9.16	-0.809	Nuclear	II
AcHB58	Acc28612	839	92363.61	5.91	-0.157	Plasma Membrane	III
AcHB59	Acc28942	293	32957.67	8.53	-0.882	Nuclear	II
AcHB60	Acc29268	720	79212.95	6.20	-0.304	Nuclear	IV
AcHB61	Acc29840	314	35485.52	4.70	-0.897	Nuclear	I
AcHB62	Acc30161	845	92393.54	6.05	-0.066	Plasma Membrane	III
AcHB63	Acc30637	272	30890.93	4.71	-0.770	Nuclear	I
AcHB64	Acc30707	260	29861.95	5.73	-0.890	Nuclear	I
AcHB65	Acc30928	260	29274.73	8.43	-0.766	Nuclear	II
AcHB66	Acc31058	211	24728.61	5.94	-0.928	Nuclear	I
AcHB67	Acc31193	200	22510.43	6.84	-0.615	Nuclear	II
AcHB68	Acc31400	323	37203.28	9.10	-0.993	Nuclear	I
AcHB69	Acc32217	844	92454.44	5.84	-0.075	Plasma Membrane	III
AcHB70	Acc33032	289	33123.77	6.23	-0.821	Nuclear	I

**Table 2:** Protein composition and physiochemical characteristics of HD-Zip proteins in Ae

Name	Genome id	Protein Length (aa)	MW (Da)	pI	GRAVY	Predicted Localiaztion	Subfamily
AeHB1	DTZ79_00g01980	260	29851.98	5.73	-0.895	Plasma Membrane	IV
AeHB2	DTZ79_01g05230	240	26759.71	6.72	-0.859	Nuclear	II
AeHB3	DTZ79_01g06050	287	33639.81	8.44	-0.853	Nuclear	II
AeHB4	DTZ79_01g09350	216	24,581.28	6.93	-0.745	Nuclear	II
AeHB5	DTZ79_01g10990	622	68506.15	5.78	-0.357	Nuclear	I
AeHB6	DTZ79_02g00740	151	17726.03	9.43	-1.099	Nuclear	I
AeHB7	DTZ79_02g09170	189	21764.88	5.14	-1.046	Nuclear	I
AeHB8	DTZ79_03g00640	345	37503.31	8.87	-0.808	Nuclear	I
AeHB9	DTZ79_03g13980	289	33120.73	6.18	-0.829	Nuclear	I
AeHB10	DTZ79_03g14000	628	68772.63	6.93	-0.202	Nuclear	I
AeHB11	DTZ79_05g01990	237	26818.90	8.72	-0.795	Nuclear	IV
AeHB12	DTZ79_05g03110	174	20120.68	9.06	-0.826	Nuclear	I
AeHB13	DTZ79_05g06340	175	19417.39	5.04	-0.600	Nuclear	I
AeHB14	DTZ79_05g09720	817	89763.83	6.19	-0.102	Nuclear	II
AeHB15	DTZ79_05g10990	813	89923.94	6.93	-0.132	Nuclear	I
AeHB16	DTZ79_06g03710	814	90154.27	5.34	-0.335	Nuclear	I
AeHB17	DTZ79_07g03650	230	25670.03	8.99	-0.658	Nuclear	I
AeHB18	DTZ79_07g09210	289	32611.42	4.70	-0.893	Nuclear	I
AeHB19	DTZ79_08g01500	745	83609.32	6.57	-0.376	Nuclear	I
AeHB20	DTZ79_08g12720	279	31246.66	8.20	-0.807	Nuclear	II
AeHB21	DTZ79_08g16270	214	25061.00	8.78	-0.967	Nuclear	IV
AeHB22	DTZ79_09g04880	289	33065.65	6.35	-0.853	Nuclear	II
AeHB23	DTZ79_11g09180	296	33203.27	9.17	-0.648	Plasma Membrane	III
AeHB24	DTZ79_12g09530	252	29170.75	8.74	-0.803	Plasma Membrane	IV
AeHB25	DTZ79_13g02300	287	33206.68	4.92	-0.870	Nuclear	I
AeHB26	DTZ79_13g02330	623	68250.91	6.26	-0.092	Nuclear	I
AeHB27	DTZ79_13g05150	183	20933.50	8.76	-0.749	Nuclear	II
AeHB28	DTZ79_13g13010	260	29022.20	6.90	-0.812	Nuclear	IV
AeHB29	DTZ79_13g13120	317	35715.79	4.83	-0.820	Chloroplast	IV
AeHB30	DTZ79_13g17160	671	74054.45	6.27	-0.303	Plasma Membrane	IV
AeHB31	DTZ79_14g02870	568	63107.30	5.39	-0.210	Nuclear	I
AeHB32	DTZ79_14g06470	263	29446.67	8.07	-0.833	Nuclear	II
AeHB33	DTZ79_15g13310	257	29881.64	8.35	-0.986	Nuclear	IV
AeHB34	DTZ79_16g02160	817	90296.99	6.80	-0.115	Plasma Membrane	III
AeHB35	DTZ79_16g07790	461	51961.41	5.96	-0.535	Nuclear	I
AeHB36	DTZ79_17g03450	679	76839.98	5.76	-0.166	Nuclear	I
AeHB37	DTZ79_18g12210	657	71,300.03	5.82	-0.083	Nuclear	III
AeHB38	DTZ79_19g14850	257	29066.34	6.85	-0.804	Nuclear	IV
AeHB39	DTZ79_20g14410	278	31439.32	8.81	-0.711	Nuclear	III
AeHB40	DTZ79_21g09440	212	24690.72	6.48	-0.873	Plasma Membrane	IV
AeHB41	DTZ79_22g05900	856	93730.17	6.28	-0.067	Nuclear	I
AeHB42	DTZ79_22g09910	838	92141.61	5.97	-0.119	Plasma Membrane	III
AeHB43	DTZ79_23g06000	311	35010.08	8.03	-0.748	Plasma Membrane	III
AeHB44	DTZ79_24g12250	268	30183.24	4.85	-0.724	Nuclear	IV
AeHB45	DTZ79_25g02120	321	35833.73	6.18	-0.774	Nuclear	II
AeHB46	DTZ79_25g07610	585	64420.94	6.00	-0.191	Nuclear	II
AeHB47	DTZ79_26g00040	785	86574.12	6.67	-0.187	Nuclear	IV
AeHB48	DTZ79_26g06750	236	26800.48	5.72	-0.936	Nuclear	I
AeHB49	DTZ79_26g09810	295	33211.85	8.22	-0.895	Plasma Membrane	III
AeHB50	DTZ79_27g02360	216	24155.18	7.62	-0.694	Nuclear	I
AeHB51	DTZ79_27g05020	314	35681.97	4.61	-0.864	Nuclear	II
AeHB52	DTZ79_27g06620	253	28048.86	5.57	-0.644	Nuclear	I
AeHB53	DTZ79_27g08380	463	50214.74	5.29	0.045	Nuclear	II
AeHB54	DTZ79_27g11080	726	79478.18	5.53	0.075	Plasma Membrane	I
AeHB55	DTZ79_29g06920	695	76801.06	6.15	-0.445	Nuclear	I

### Phylogenetic reconstruction of kiwifruit HD-Zips

**Fig. 2:**
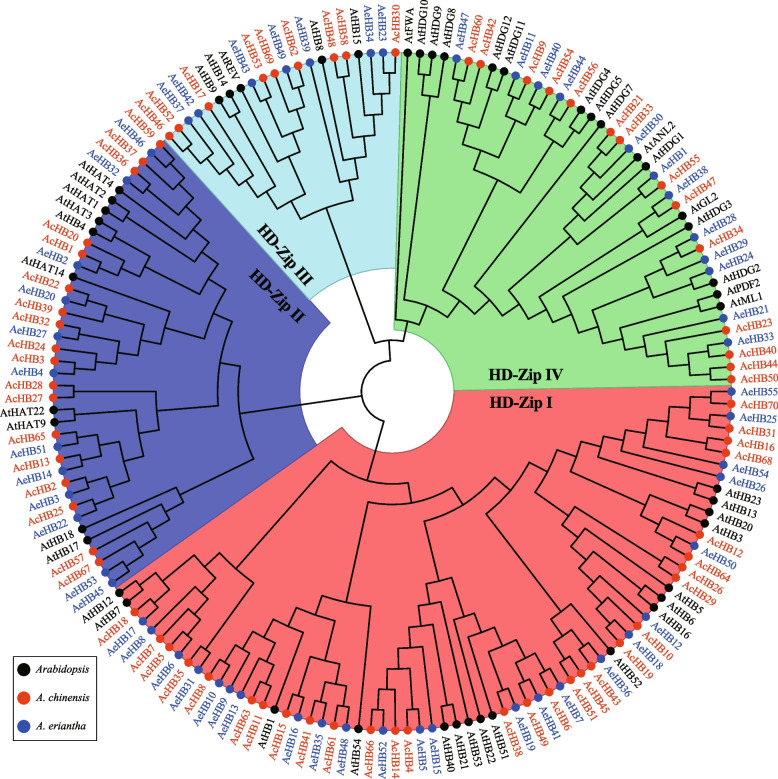
Phylogenetic tree of HD-Zip proteins. The full-length HD-Zip protein sequences from Arabidopsis (At, black gene name and circles), *A. chinensis* (Ac, red gene name and circles), and *A. eriantha* (Ae, blue gene name, and circles) were aligned using ClustalX 2.0 with default parameters. The unrooted phylogenetic tree was constructed using MEGA X and the Neighbor-Joining method. Subfamily I to IV were highlighted using red, blue, light green, and green sectors, respectively

To investigate the phylogenetic relationships of *HD-Zip* genes in the two kiwifruit species, we conducted a neighbor-joining (NJ) tree analysis using the full-length protein sequences of the identified 70 AcHBs, 55 AeHBs, and 48 AtHD-Zip*s*. The results revealed that both AcHBs and AeHBs were classified into four subfamilies (HD-Zip I, HD-Zip II, HD-Zip III, and HD-Zip IV), which was consistent with previous findings in Arabidopsis and other species [28–31] (Fig. 2). Subfamily I had the most AcHBs (29) and AeHBs (24), while subfamily III had the least AcHBs (9) and AeHBs (7) (Fig. 2, Tables 1 and 2). Subfamily II contained 18 AcHBs and 12 AeHBs, while subfamily IV had 14 AcHBs and 12 AeHBs (Fig. 2, Tables 1 and 2). Both AcHBs and AeHBs grouped with different *HD-Zip* genes in Arabidopsis, indicating that they probably possessed functional diversifications similar to *HD-Zip* genes in Arabidopsis and other species (Fig. 2).

### Chromosomal distribution and gene structure of kiwifruit HD-Zips

**Fig. 3:**
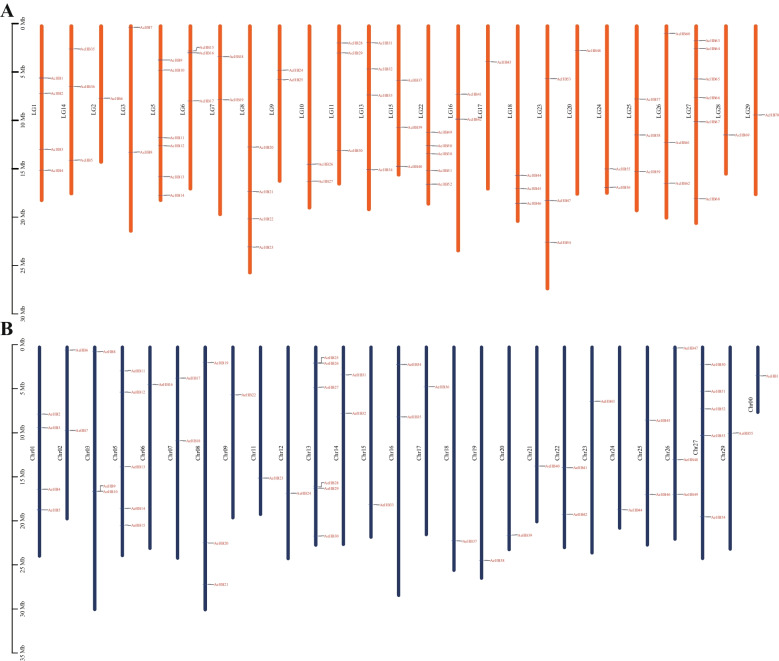
Distribution of HD-Zip genes in Ac **A** and Ae **B** genomes

The 70 *AcHB* genes were distributed randomly across 25 chromosomes of Ac, with chromosome 5 and 27 having the highest number of *AcHB* genes, each containing 6 genes, followed by chromosome 22 with 5 genes (Fig. 3A and Table S1). Chromosomes 1, 8, and 13 had 4 *AcHB* genes, while chromosomes 6, 11, 14, 15, 18, 23, 25, and 26 had 3, and chromosomes 3, 7, 9, 10, 16, and 24 had 2 (Fig. 3A and Table S1). The remaining five chromosomes (chromosomes 2, 17, 20, 28, and 29) had one *AcHB* gene each (Fig. 3A and Table S1). Similarly, the 55 *AeHB* genes were unevenly distributed across 26 chromosomes and one contig (chromosome 00), with chromosome 13 having the highest number of *AeHB* genes (6) (Fig. 3B and Table S1). Chromosomes 5 and 27 had 5 *AeHB* genes, chromosome 1 had 4, while chromosomes 3, 8, and 26 had 3, and chromosomes 2, 7, 14, 16, 22, and 25 had 2 (Fig. 3B and Table S1). The remaining 13 chromosomes had one *AeHB* gene each (Fig. 3B and Table S1).

**Fig. 4:**
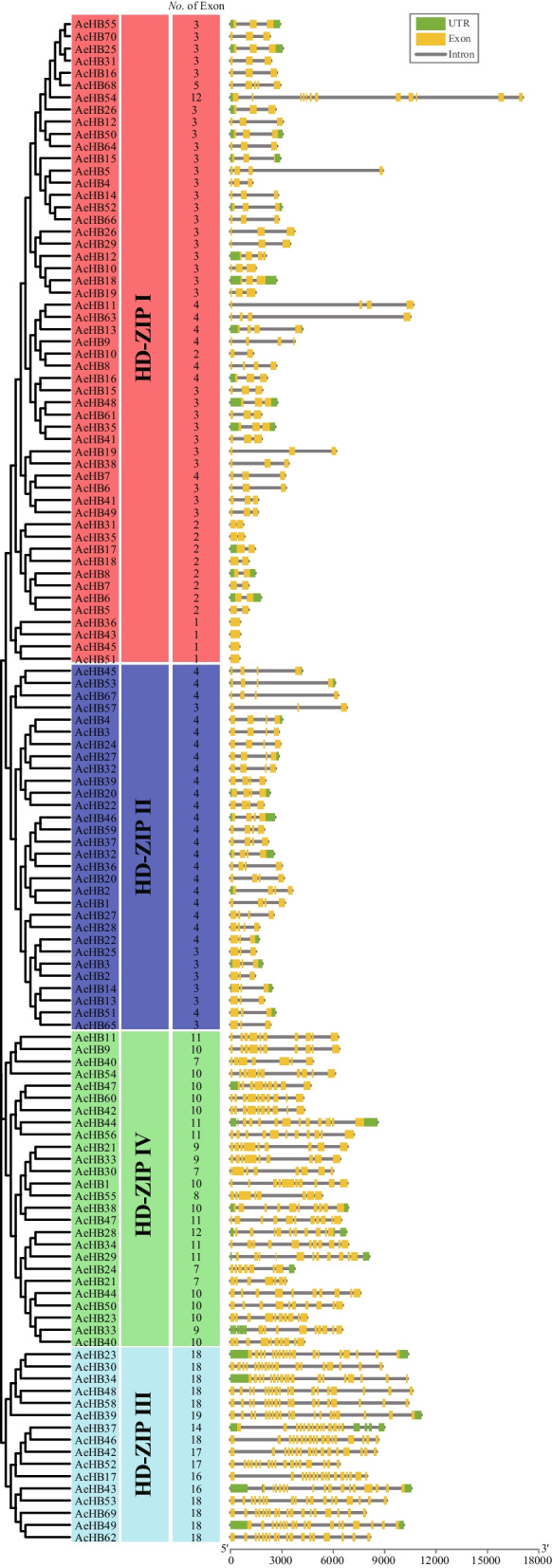
Exon–intron structures of HD-Zip genes in two kiwifruit species. The left panel indicated the phylogenetic tree containing AcHB and AeHB proteins; the middle panel showed the ranges of four clades; the right panel showed exon–intron structures of kiwifruit HD-Zip genes. The green rectangle shows exons, the yellow rectangle shows UTRs, and the regular line represents introns

The gene structure is an important evolutionary feature of a gene family and provides insights into their function diversification and classification. The number of exons in *AcHBs* and *AeHBs* ranged from 1 to 19 (Fig. 4). However, the number of exons in *AcHBs* and *AeHBs* belonging to different subfamilies varied greatly (Fig. 4 and Fig. S1). The average number of exons in subfamilies I, II, III, and IV were 3.01, 3.76, 17.43, and 9.65, respectively (Fig. S1). Most *AcHB* and *AeHB* genes grouped in the same clade had a similar exon–intron organization (Fig. 4).

### Conserved domain analysis and motif composition of kiwifruit HD-Zip

**Fig. 5:**
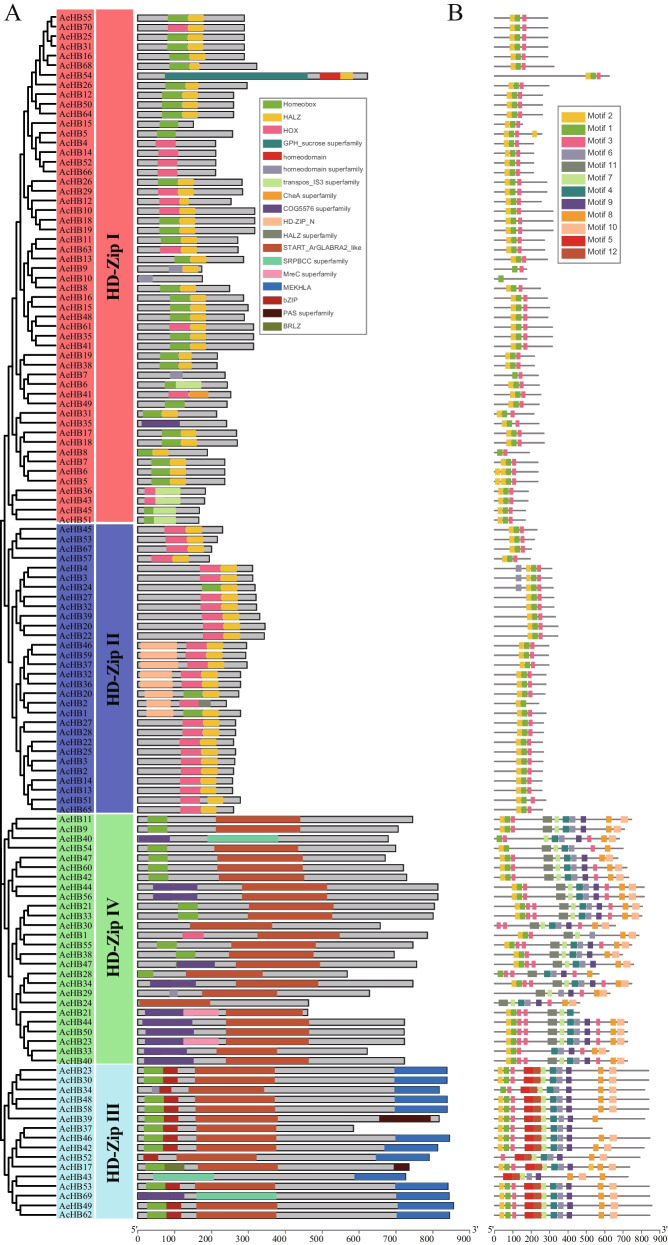
Conserved domain and motif architectures of kiwifruit HD-Zip proteins. **A** The first panel indicated the phylogenetic tree of AcHB and AeHB protein sequences; the second panel showed the defined clades; the third panel showed conserved domain architectures; **B** The motif architectures of HD-Zip proteins. Rectangles with different colors represented different conserved domains and motifs

Conserved domains are essential functional elements of proteins, and we identified the conserved domains of kiwifruit HD-Zips to infer their potential functions and functional diversification. Our results showed that the conserved domain architectures of kiwifruit HD-Zips belonging to the same subfamily were more similar than those belonging to different subfamilies (Fig. 5A). In addition to the homeodomain and LZ domain, kiwifruit HD-Zips also harbored several other conserved domains, indicating functional diversification (Fig. 5A). Five *AcHBs* and three *AeHBs* belonging to the HD-Zip II subfamily contained the HD-Zip protein N-terminus domain (PF04618) with unknown functions (Fig. 5A). All kiwifruit HD-Zips grouped into the HD-Zip III and IV subfamilies contained the START domain, which was consistent with results in other species (Fig. 5A) [8, 20]. Moreover, the HD-Zip III subfamily possessed the MEKHLA domain (Fig. 5A).

To infer the potential functions and functional diversification of kiwifruit *HD-Zips*, we identified their conserved domains and motifs. Conserved domains are typically functional elements of proteins, and our results showed that the conserved domain architectures of kiwifruit HD-Zips belonging to the same subfamily were more similar than those belonging to different subfamilies. In addition to the homeodomain and LZ domain, kiwifruit HD-Zips contained several other conserved domains, indicating their functional diversification. For example, some AcHBs and AeHBs in the HD-Zip II subfamily harbored the HD-Zip protein N-terminus domain with unknown functions, while all kiwifruit HD-Zips in the HD-Zip III and IV subfamilies contained the START domain, consistent with previous findings in other species. We also used MEME software to predict the motif compositions of kiwifruit HD-Zips and identified 12 conserved motifs (Fig. 5B and Fig. S2). The motif numbers of kiwifruit HD-Zips belonging to different subfamilies were significantly different, indicating their different motif organizations (Fig. S3). Almost all kiwifruit HD-Zips contained motif 1–3, which spanned the homeodomain and LZ domain (Fig. 5), while subfamily-specific motifs were also identified. Consistent with the results of the exon–intron structure, kiwifruit HD-Zips showing a closer phylogenetic relationship had more similar conserved motif structures, indicating similar functions.

### Synteny analysis of kiwifruit HD-Zips

**Fig. 6:**
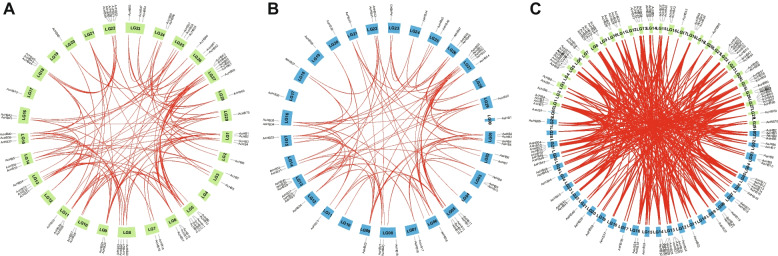
Chromosome distribution and synteny relationship of HD-Zip genes in two kiwifruit species. The green and blue bars indicated chromosomes for Ac and Ae, respectively. The syntenic gene pairs were connected by lines with different colors. **A** Ac, **B** Ae, **C** Ac-Ae

Gene duplication and loss are key evolutionary forces that contribute to the expansion or contraction of gene families. Duplicated genes can lead to either gene redundancy or new functionalization. To explore the evolutionary history of kiwifruit *HD-Zip* genes, we conducted synteny analysis between the two kiwifruit species. We visualized the locus relationship of homologous *HD-Zip* genes and gene duplication events using MCScanX [32]. In the study of Ac, a total of 73 gene duplication events were identified. Similarly, in the analysis of Ae, 34 gene duplication events were discerned (Fig. 6 and Table 3). Interestingly, we found that duplicated gene pairs were randomly distributed across all subfamilies (Fig. 6 and Table 3). Furthermore, all duplicated gene pairs were produced by whole-genome duplication (WGD) or segmental, indicating that WGD or segmental has played a significant role in the expansion of kiwifruit *HD-Zips* compared to HD-Zips in *Arabidopsis thaliana* (Table 3).

**Table 3:** HD-Zip duplication events identified in kiwifruit

Species	Duplicate pairs	Ka	Ks	Ka/Ks	Duplication	Duplication date (Mya)
*A. chinensis*	AcHB1/AcHB36	0.220	1.420	0.155	WGD	47.33
	AcHB1/AcHB20	0.068	0.235	0.289	WGD	7.85
	AcHB2/AcHB27	0.334	1.698	0.197	WGD	56.59
	AcHB2/AcHB28	0.310	1.989	0.156	WGD	66.30
	AcHB2/AcHB65	0.140	0.767	0.183	WGD	25.58
	AcHB2/AcHB13	0.138	0.852	0.162	WGD	28.41
	AcHB2/AcHB25	0.028	0.226	0.122	WGD	7.55
	AcHB3/AcHB32	0.214	0.792	0.270	WGD	26.40
	AcHB3/AcHB24	0.072	0.198	0.366	WGD	6.59
	AcHB4/AcHB66	0.158	0.845	0.187	WGD	28.18
	AcHB4/AcHB14	0.146	1.070	0.136	WGD	35.66
	AcHB5/AcHB7	0.072	0.185	0.392	WGD	6.15
	AcHB6/AcHB49	0.082	0.215	0.379	WGD	7.18
	AcHB7/AcHB18	0.391	2.696	0.145	WGD	89.85
	AcHB10/AcHB19	0.041	0.194	0.212	WGD	6.48
	AcHB12/AcHB16	0.267	1.072	0.249	WGD	35.74
	AcHB13/AcHB25	0.139	0.716	0.194	WGD	23.86
	AcHB26/AcHB29	0.069	0.158	0.435	WGD	5.27
	AcHB27/AcHB28	0.047	0.175	0.266	WGD	5.84
	AcHB27/AcHB65	0.312	1.512	0.206	WGD	50.41
	AcHB27/AcHB13	0.299	1.929	0.155	WGD	64.31
	AcHB27/AcHB25	0.328	1.368	0.240	WGD	45.58
	AcHB28/AcHB65	0.303	1.284	0.236	WGD	42.81
	AcHB28/AcHB13	0.298	1.443	0.206	WGD	48.10
	AcHB28/AcHB25	0.301	1.471	0.205	WGD	49.02
	AcHB30/AcHB48	0.091	0.996	0.091	WGD	33.20
	AcHB30/AcHB58	0.089	0.957	0.093	WGD	31.88
	AcHB31/AcHB68	0.171	0.880	0.195	WGD	29.34
	AcHB31/AcHB64	0.205	2.075	0.099	WGD	69.17
	AcHB31/AcHB70	0.016	0.126	0.129	WGD	4.21
	AcHB31/AcHB12	0.220	1.557	0.141	WGD	51.91
	AcHB31/AcHB16	0.097	0.926	0.105	WGD	30.85
	AcHB32/AcHB24	0.226	0.773	0.292	WGD	25.77
	AcHB33/AcHB21	0.021	0.310	0.067	WGD	10.32
	AcHB34/AcHB40	0.163	2.704	0.060	WGD	90.12
	AcHB35/AcHB7	0.461	1.435	0.321	WGD	47.83
	AcHB35/AcHB18	0.449	2.219	0.202	WGD	73.96
	AcHB36/AcHB37	0.126	0.726	0.173	WGD	24.19
	AcHB36/AcHB59	0.130	0.688	0.189	WGD	22.92
	AcHB36/AcHB20	0.335	1.365	0.246	WGD	45.50
	AcHB37/AcHB59	0.021	0.143	0.150	WGD	4.75
	AcHB37/AcHB20	0.247	1.550	0.159	WGD	51.68
	AcHB39/AcHB59	0.319	2.012	0.158	WGD	67.05
	AcHB39/AcHB22	0.043	0.268	0.161	WGD	8.94
	AcHB40/AcHB44	0.066	0.793	0.083	WGD	26.44
	AcHB40/AcHB50	0.073	0.812	0.090	WGD	27.05
	AcHB40/AcHB23	0.031	0.146	0.213	WGD	4.86
	AcHB41/AcHB61	0.036	0.153	0.234	WGD	5.10
	AcHB41/AcHB15	0.331	2.083	0.159	WGD	69.43
	AcHB42/AcHB60	0.039	0.141	0.278	WGD	4.69
	AcHB44/AcHB50	0.027	0.175	0.155	WGD	5.83
	AcHB44/AcHB23	0.065	0.809	0.080	WGD	26.96
	AcHB45/AcHB51	0.050	0.162	0.308	WGD	5.39
	AcHB46/AcHB52	0.029	0.177	0.163	WGD	5.89
	AcHB48/AcHB58	0.022	0.113	0.193	WGD	3.75
	AcHB53/AcHB62	0.054	0.453	0.120	WGD	15.11
	AcHB53/AcHB69	0.052	0.441	0.119	WGD	14.70
	AcHB54/AcHB60	0.453	2.059	0.220	WGD	68.63
	AcHB55/AcHB21	0.241	3.356	0.072	WGD	111.88
	AcHB57/AcHB67	0.030	0.171	0.176	WGD	5.70
	AcHB59/AcHB20	0.236	1.196	0.198	WGD	39.87
	AcHB61/AcHB15	0.365	1.628	0.224	WGD	54.27
	AcHB62/AcHB69	0.019	0.127	0.149	WGD	4.24
	AcHB63/AcHB11	0.046	0.217	0.212	WGD	7.24
	AcHB64/AcHB70	0.197	1.697	0.116	WGD	56.57
	AcHB64/AcHB12	0.036	0.209	0.174	WGD	6.96
	AcHB64/AcHB16	0.252	1.597	0.158	WGD	53.24
	AcHB65/AcHB13	0.032	0.214	0.152	WGD	7.12
	AcHB65/AcHB25	0.158	0.656	0.241	WGD	21.85
	AcHB66/AcHB14	0.064	0.195	0.327	WGD	6.49
	AcHB68/AcHB70	0.181	0.813	0.223	WGD	27.09
	AcHB68/AcHB16	0.073	0.158	0.459	WGD	5.28
	AcHB70/AcHB16	0.106	0.851	0.125	WGD	28.36
*A. eriantha*	AeHB3/AeHB14	0.155	0.845	0.184	WGD	28.17
	AeHB3/AeHB22	0.034	0.211	0.163	WGD	7.04
	AeHB3/AeHB51	0.141	0.700	0.202	WGD	23.34
	AeHB4/AeHB27	0.216	0.813	0.266	WGD	27.10
	AeHB5/AeHB15	0.139	0.955	0.146	WGD	31.85
	AeHB5/AeHB52	0.184	0.931	0.197	WGD	31.03
	AeHB6/AeHB8	0.070	0.211	0.332	WGD	7.04
	AeHB7/AeHB41	0.152	0.288	0.527	WGD	9.61
	AeHB8/AeHB17	0.368	1.484	0.248	WGD	49.48
	AeHB8/AeHB31	0.422	1.225	0.344	WGD	40.83
	AeHB9/AeHB13	0.149	0.701	0.213	WGD	23.37
	AeHB14/AeHB22	0.148	0.769	0.193	WGD	25.62
	AeHB14/AeHB51	0.042	0.237	0.178	WGD	7.91
	AeHB15/AeHB52	0.126	0.276	0.459	WGD	9.19
	AeHB16/AeHB35	0.326	1.388	0.235	WGD	46.27
	AeHB16/AeHB48	0.316	1.249	0.253	WGD	41.63
	AeHB16/AeHB50	0.542	2.289	0.237	WGD	76.30
	AeHB17/AeHB31	0.420	1.877	0.224	WGD	62.56
	AeHB21/AeHB24	0.267	2.158	0.124	WGD	71.94
	AeHB21/AeHB33	0.057	0.229	0.248	WGD	7.64
	AeHB22/AeHB51	0.148	0.577	0.257	WGD	19.23
	AeHB23/AeHB34	0.034	0.152	0.226	WGD	5.07
	AeHB24/AeHB28	0.176	0.939	0.188	WGD	31.29
	AeHB24/AeHB33	0.217	2.306	0.094	WGD	76.86
	AeHB25/AeHB50	0.203	2.236	0.091	WGD	74.54
	AeHB25/AeHB55	0.018	0.120	0.148	WGD	4.00
	AeHB29/AeHB33	0.251	3.008	0.084	WGD	100.26
	AeHB32/AeHB46	0.138	0.639	0.216	WGD	21.29
	AeHB35/AeHB48	0.042	0.156	0.271	WGD	5.20
	AeHB37/AeHB42	0.055	0.246	0.224	WGD	8.21
	AeHB43/AeHB49	0.067	0.436	0.154	WGD	14.53
	AeHB45/AeHB53	0.040	0.254	0.157	WGD	8.46
	AeHB50/AeHB54	0.279	1.315	0.212	WGD	43.84
	AeHB50/AeHB55	0.190	1.561	0.121	WGD	52.02

To investigate the evolutionary forces that drove the expansion or contraction of kiwifruit *HD-Zip* gene families, we conducted synteny analysis of *HD-Zip* genes in both kiwifruit species using MCScanX. We identified a total of 73 and 34 gene duplication events in Ac and Ae, respectively (Fig. 6 and Table 3). These events were randomly distributed among all subfamilies and were produced by whole-genome duplication (WGD) or segmental, indicating that WGD or segmental was the primary driver of *HD-Zip* gene expansion in kiwifruit. To estimate the selection pressure experienced by duplicated genes, we calculated the ratios of nonsynonymous (Ka) versus synonymous (Ks) substitution rates for each duplicated gene pair. The Ka/Ks values ranged from 0.060–0.459 and 0.084–0.527 for Ac and Ae, respectively (Table 3). All duplicated gene pairs exhibited Ka/Ks values less than one, indicating that the duplicated genes were under purifying selection and that their potential functions were conserved.

### Cis-element analysis of promoter regions of kiwifruit HD-Zips

*Cis*-elements play a crucial role in transcriptional regulation and significantly impact gene function. We extracted the 2000-bp upstream region of each kiwifruit *HD-Zip* gene and used it to predict the *cis*-elements. We identified 19 functional cis-elements, including core promoter elements such as TATA-box and CAAT-box, in the promoter regions of kiwifruit *HD-Zips*. These *cis*-elements were classified into four subfamilies, including light responsiveness, plant growth and development, hormone-responsive, and stress-responsive subfamily (Figs. S4 and S5). The plant growth and development subfamily was the most abundant within the promoter regions of kiwifruit *HD-Zip* genes, suggesting that kiwifruit *HD-Zips* play a significant role in regulating kiwifruit growth and development (Fig. S5). Overall, the number of *cis*-elements in the Ae *HD-Zip* promoter was lower than that in the Ac *HD-Zip* promoter (Fig. S5). The *cis*-element arrangements for the duplicated gene pairs listed in Table 3 were divergently evolved, suggesting specific expression patterns and new functionalization for the duplicated gene pairs (Fig. S4). However, the *cis*-element arrangements of the orthologous *HD-Zip* gene pairs for the two species had high similarities, indicating that the orthologous *HD-Zip* gene pairs possessed similar functions (Fig. S4).

### Expression patterns of kiwifruit HD-Zips

**Fig. 7:**
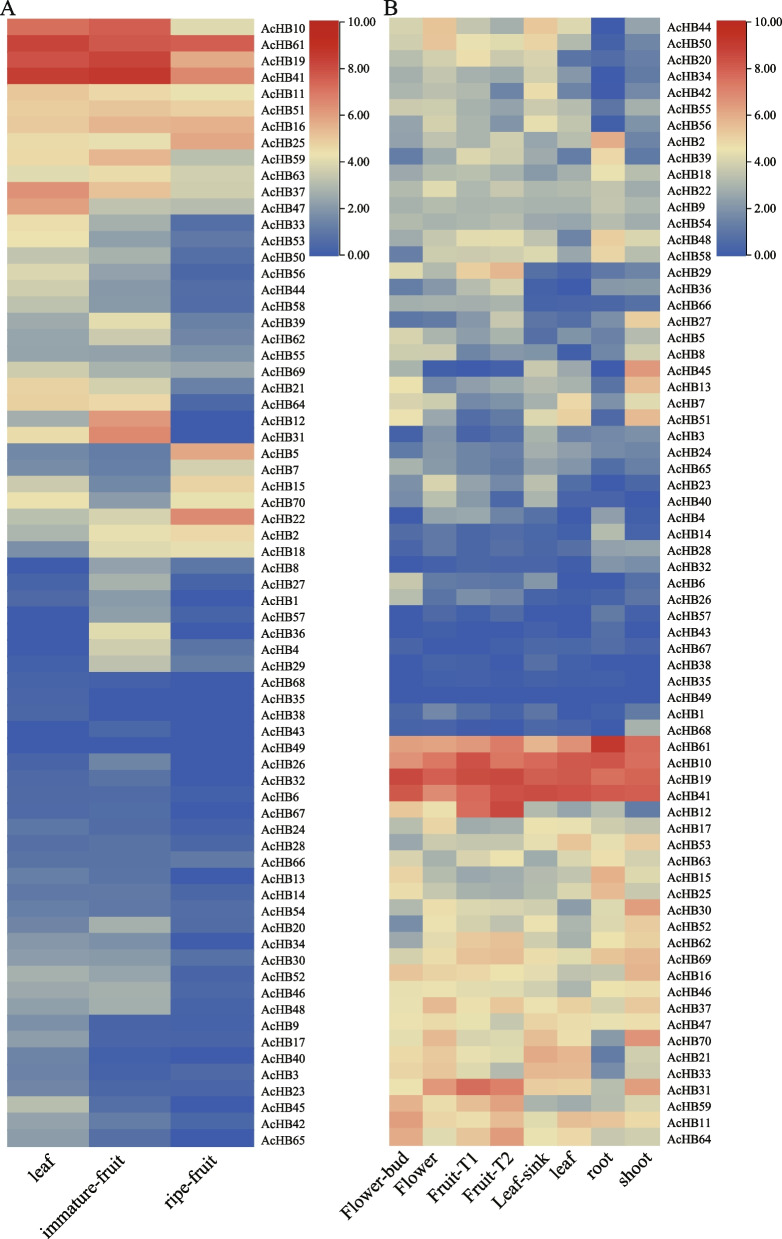
Expression profiles of AcHB genes in different tissues. The heatmap indicated log2 rate values of the FPKM (fragments per kilobase of exon model per million mapped reads) values of AcHB genes. **A** Expression profiles of *AcHBs* in three tissues. **B** Expression profiles of AcHB in seven tissues

To investigate the expression patterns of *AcHB* genes in different tissues, we collected two transcriptome datasets (Fig. 7A). The first dataset compared the expression profiles of three tissues: leaf, immature fruit, and ripe fruit. The expression patterns of *HD-Zip* genes in Ac could be classified into three groups. The first group included most *HD-Zip* genes, which exhibited low expression levels in all three tissues. The expression patterns of *HD-Zip* genes in the second group showed high tissue-specificity. For example, *AcHB37* and *AcHB47* were highly expressed in kiwifruit leaf, while three genes (*AcHB12/31/59*) were expressed in immature kiwifruit fruit, and three other genes (*AcHB5/22/25*) were highly expressed in ripe kiwifruit (Fig. 7A). Four *HD-Zip* genes (*AcHB10/19/41/61*) were expressed in all three tissues (Fig. 7A). The second transcriptome dataset investigated the expression profiles of eight tissues and showed that different *HD-Zip* family members exhibited divergent expression patterns in different tissues (Fig. 7B). Four *HD-Zip* genes (*AcHB10/19/41/61*) were expressed in all eight tissues, indicating their essential role in kiwifruit development (Fig. 7B). The tissue-specific expression patterns of *HD-Zip* genes in Ac illustrate gene function diversification.

**Fig. 8:**
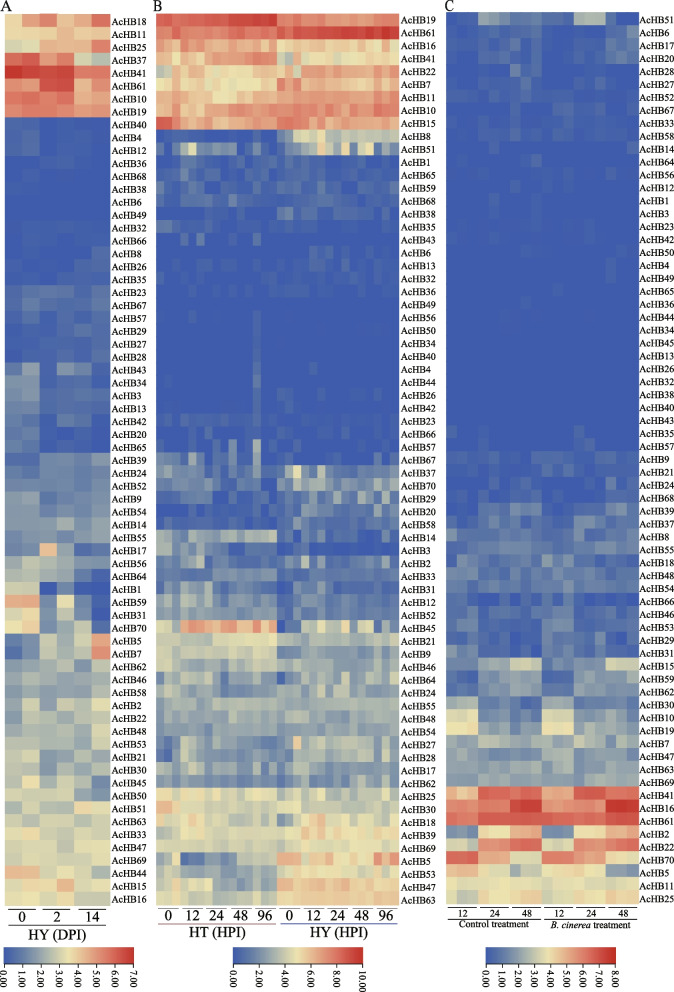
Expression profiles of *AcHB* genes with invasions of different pathogens. **A** Expression profiles of AcHBs in the susceptible cultivars 'HY' to Psa invasion. DPI, days post-infection. **B** Expression profiles of AcHBs in two kiwifruit cultivars infected with Psa. HT and HY represented resistant and susceptible cultivars, respectively. The number following the cultivar name showed hours post the Psa invasion (HPI). **C** Expression profiles of AcHBs in kiwifruit cultivar 'HY' infected with *B. cinerea*. The number following the cultivar name showed hours post the Psa invasion (HPI)

To investigate the potential role of the *HD-Zip* gene family in regulating kiwifruit resistance or tolerance to pathogen invasion, we analyzed three transcriptome datasets (Fig. 8). In the first dataset, we compared the transcriptional responses of the susceptible cultivars 'hongyang' (HY) to the invasion of *Pseudomonas syringae* pv. *actinidiae* (Psa) (Fig. 8A). We found that the expression patterns of several *HD-Zip* genes were significantly altered upon Psa invasion, such as *AcHB25/37* (Fig. 8A). In the second dataset, we compared the expression profiles of two kiwifruit materials with different resistance levels to Psa, namely HT (highly resistant) and HY (susceptible) (Fig. 8B). We divided the expression patterns of *HD-Zip* genes into four clades and found that expression levels of multiple *HD-Zip* genes were significantly altered with the invasion of Psa, such as *AcHB19/61* (Fig. 8B). We also found that *AcHB45* positively regulated kiwifruit resistance/tolerance to Psa, as its expression level was increased in HT but decreased in HY (Fig. 8B). On the other hand, *AcHB5/47* had the opposite effect, indicating that they negatively regulated kiwifruit resistance/tolerance to Psa (Fig. 8B). In the third transcriptome dataset, we investigated kiwifruit responses to the infection of *Botrytis cinerea* (Fig. 8C). Consistent with the results from the first and second dataset, we found that *AcHB61* had a high expression level upon *Botrytis cinerea* infection (Fig. 8C). These results suggest that *HD-Zip* genes play an important role in regulating kiwifruit responses to pathogen invasion.

**Fig. 9:**
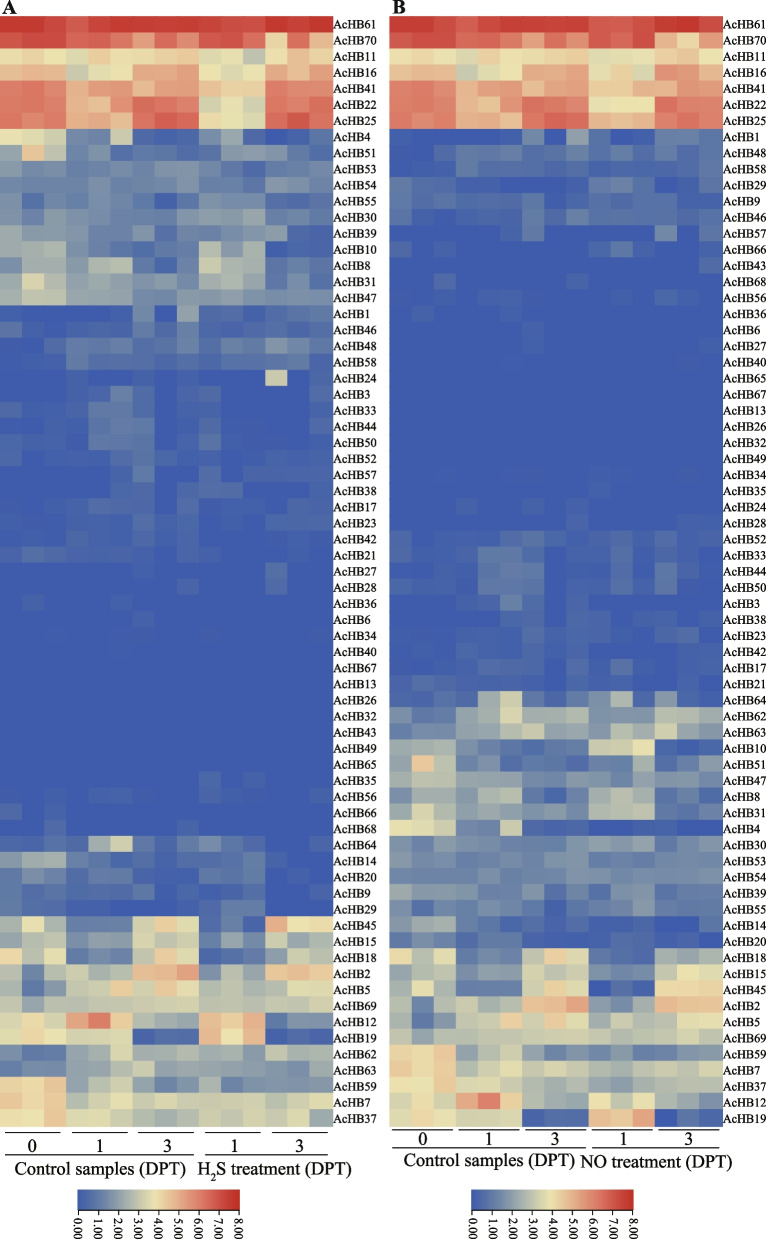
Expression profiles of *AcHB* genes with stress treatment. The number following the cultivar name showed days post the treatment (DPT). **A** Expression profiles of AcHBs with the Hydrogen sulfide (H_2_S) treatment, **B** Expression profiles of AcHBs with the Nitric oxide (NO) treatment

We further investigated the potential role of *HD-Zips* in regulating postharvest processes of kiwifruit using two transcriptome datasets. Previous research has shown that hydrogen sulfide (H_2_S) can delay the maturation of kiwifruit [33], and the first transcriptome profile estimated kiwifruit responses to the H_2_S treatment. We found that the H_2_S treatment changed the expression levels of *HD-Zip* genes (Fig. 9A). The expression level of *AcHB19* was increased after one day of the H_2_S treatment, suggesting that *AcHB19* played a role in delaying the maturation of kiwifruit (Fig. 9a). The expression profiles of three *HD-Zip* genes (*AcHB22/25/41*) were reduced after one day of the H_2_S treatment, indicating that those *HD-Zips* accelerated the maturation of kiwifruit (Fig. 9A). Nitric oxide (NO) is an important signal molecule in regulating the ripening of kiwifruit [34], and the second transcriptome data investigated the expression profiles of kiwifruit in response to the NO treatment (Fig. 9B). Similar to the results of the H_2_S treatment, the NO treatment altered the expression levels of *HD-Zip* genes (Fig. 9B). For example, the expression level of *AcHB19* was increased, and the expression profiles of three *HD-Zip* genes (*AcHB22/25/41*) were reduced (Fig. 9B).

### Suppression of multiple AcHB Genes expression by Psa infection

**Fig. 10:**
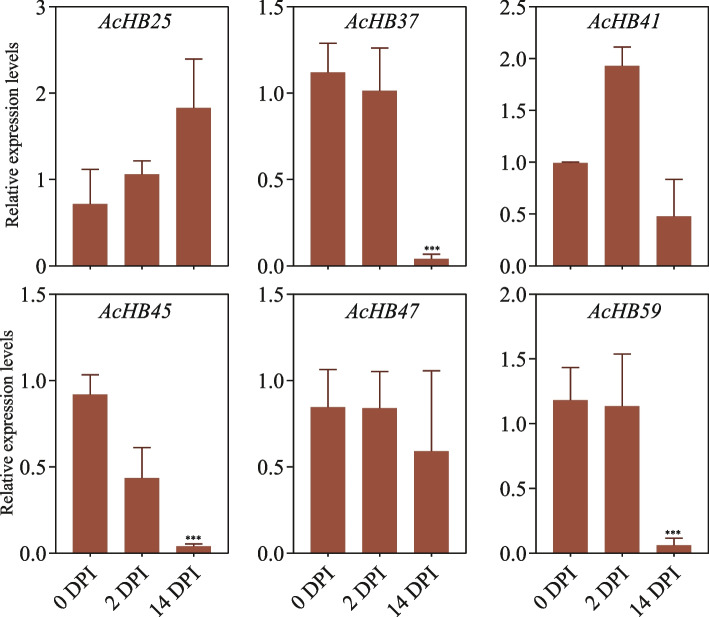
Expression analysis of *AcHB*s using RT-qPCR at different times with Psa infection. Actin was used as the internal standard for each gene. DPI, days post incubation

In order to elucidate and verify the involvement of the *AcHB* gene family in response to kiwifruit bacterial canker pathogen (*Pseudomonas syringae* pv. *actinidiae*, Psa) infection, an inoculation experiment for bacterial canker was conducted. Concurrently, leveraging transcriptomic data (Fig. 9), six differentially expressed genes were selected for validation through fluorescent quantitative PCR. Among these, three AcHB genes (*AcHB37/45/59*) exhibited a significant reduction in expression levels at day 14 post Psa infection (*p*-value < 0.001, Fig. 10), highlighting their role in the kiwifruit's response to Psa infection. These three genes emerge as crucial candidates for subsequent gene functional studies and mechanistic investigations.

## Discussion

The HD-Zip gene family is pivotal in modulating various facets of plant growth, development, and stress response mechanisms [1–3, 5]. While the HD-Zip gene family has been recognized in multiple plant species [5, 6, 28–31], a comprehensive genome-wide analysis of this gene family in kiwifruit remains unexplored. In our research, we undertook a comprehensive genome-wide identification of the HD-Zip gene family in two distinct kiwifruit species: Ac and Ae. We conducted a comparative analysis of HD-Zip characteristics between these species and examined the organization of cis-elements within the promoter regions of the identified HD-Zips across both species. Furthermore, we probed the expression patterns of Ac HD-Zips across varied tissues and under stress conditions.

We detected 70 and 55 HD-Zip genes in Ac and Ae, respectively (Fig. 1, Tables 1 and 2). These numbers were higher than the number of HD-Zip family members in Arabidopsis (48 members), indicating that the HD-Zip gene family expanded in kiwifruit. Synteny analysis showed that the duplicated HD-Zip gene pairs in both species were all caused by whole-genome duplication (WGD) or segmental events (Fig. 6 and Table 3). Our genomic analyses also confirmed that three ancient WGD or segmental events occurred in both Ac and Ae genomes (Table 3) [23, 26]. However, there was a significant difference in the number of HD-Zip genes between Ac and Ae, suggesting that the HD-Zip gene family evolved differently in these two species, which is consistent with previous genomic analyses [23, 26]. We suggest that the differences in the number and distribution of HD-Zip genes in Ac and Ae may be due to translocation, gene retention, and loss patterns after WGD or segmental. All HD-Zip genes in both species were under purifying selection (Table 3), indicating that these genes are important for kiwifruit development and adaptation. Similar to other species, we divided the kiwifruit HD-Zip gene family into four clades (subfamily I to IV) based on phylogenetic analysis (Fig. 2). We further analyzed the expression profiles of AcHDZips in different tissues and under stress treatments, revealing their potential functions in regulating kiwifruit growth, development, and stress responses.

Our study explored the functional diversification of kiwifruit HD-Zips through analysis of conserved motifs, cis-elements, and expression patterns. In addition to the homeodomain and LZ domain, we found several other conserved domains within kiwifruit HD-Zips, indicative of functional diversification (Fig. 5A). We identified subfamily-specific conserved domains, including the START and MEKHLA domains (Fig. 5A). Furthermore, we observed clade-specific or subclade-specific motifs, suggesting a functional differentiation among HD-Zip genes from different clades (Fig. 5B). However, gene structures and conserved motif organizations were largely consistent across most kiwifruit HD-Zips from the same subclade. A cis-element analysis of the promoter regions of these HD-Zips revealed significant variation in cis-element organization within the same subclade (Fig. S4). We propose that the cis-element organization of HD-Zips from the same subclade regulates their functional divergence by controlling their expression patterns, a hypothesis supported by our expression analysis results (Figs. 7, 9, and 10). Overall, our findings suggest that gene structure, motif organization, and cis-element arrangement play a crucial role in regulating the functional diversification of kiwifruit HD-Zips.

## Conclusions

In conclusion, our study provides a comprehensive characterization of the homeodomain-leucine zipper (HD-Zip) gene family in kiwifruit. We systematically identified and categorized 70 HD-Zip genes in Actinidia chinensis (Ac) and 55 in Actinidia eriantha (Ae), classifying them into four subfamilies (HD-Zip I, II, III, and IV) through rigorous phylogenetic analysis. Insightful analyses of synteny patterns and selection pressures highlighted the potential contributions of whole-genome duplication (WGD) or segmental events to the divergence in gene numbers between the two kiwifruit species, with duplicated gene pairs undergoing purifying selection. Additionally, our investigation unveiled tissue-specific expression patterns among kiwifruit HD-Zip genes, identifying certain genes as crucial regulators of responses to bacterial canker disease and postharvest processes. These findings not only enhance our understanding of the evolutionary and functional aspects of kiwifruit HD-Zips but also illuminate their roles in plant growth and development.

## Materials and methods

### HD-Zip gene identification in two kiwifruit species

To identify candidate genes of the HD-Zip family in Ac and Ae genomes, we utilized the Hidden Markov Model (HMM) of the HD-domain profile (PF00046) and LZ domain profile (PF02183) through the software HMMER 3.0 [27]. We obtained the whole-genome sequences and protein sequences of both kiwifruit species from the Kiwifruit Genome Database (http://kiwifruitgenome.org/) and collected all HD-Zip protein sequences of *Arabidopsis* from the TAIR website (https://www.arabidopsis.org/). To confirm the presence of the homeodomain and LZ domain in the candidate HD-Zip proteins, we employed the Conserved Domain Database (CDD) (https://www.ncbi.nlm.nih.gov/Structure/cdd/cdd.shtml) and the simple modular architecture research tool (SMART) (http://smart.embl.de/). Only candidate HD-Zip proteins that contained both the homeodomain and the LZ domain were used for further analysis.

### Physicochemical properties analysis of kiwifruit HD-Zip

To further analyze the characteristics of the HD-Zip gene family in the two kiwifruit species, we computed their physicochemical properties, including protein length, theoretical isoelectric point (pI), grand average of hydropathicity (GRAVY), and molecular weight (MW). These properties were calculated using the ProtParam tool available on the ExPASy server (http://web.expasy.org/protparam/). Additionally, we predicted the subcellular localization of kiwifruit HD-Zip proteins using the online software CELLO (v2.5, http://cello.life.nctu.edu.tw/).

### Gene structure, motif analysis, and chromosomal distribution of kiwifruit HD-Zip

The genomic and coding sequences of HD-Zip genes in both kiwifruit species (*A. chinensis* and *A. eriantha*) were obtained using TBtools. The gene structures were then visualized using the Gene Structure Display Server (GSDS 2.0). To identify the conserved motifs of HD-Zip proteins, the Multiple Expectation Maximization for Motif Elicitation tool (MEME) was used with a maximum of 12 motifs. The genome locations of HD-Zip genes were extracted from the corresponding GFF file using a Perl script, and the chromosomal distributions were illustrated using MapGene2 Chrome (http://mg2c.iask.in/mg2c_v2.0/).

### Construction of phylogenetic tree for kiwifruit HD-Zip proteins

We retrieved the full-length protein sequences of HD-Zip genes from *Arabidopsis thaliana*, *A. chinensis*, and *A. eriantha* and performed multiple sequence alignments using ClustalX with default parameters [35]. The resulting aligned sequences were used to construct a phylogenetic tree using the neighbor-joining (NJ) method with a bootstrap value of 3000 in MEGA X software [36].

### Syntenic analysis and duplication events identification of kiwifruit HD-Zip

To investigate the syntenic relationship and gene duplication of kiwifruit HD-Zip proteins, we retrieved all protein sequences of Ac and Ae and performed BLASTP alignment with an e-value of 1 × 10–10. We then identified syntenic relationships and duplication patterns of kiwifruit HD-Zip using the MCScanX software with default parameters [29]. The synonymous (Ks) and nonsynonymous (Ka) mutation rates of the duplicated HD-Zip gene pairs were computed using TBtools software [30]. To produce collinearity blocks across the whole genome, we conducted syntenic analysis of kiwifruit HD-Zip using the MCScanX software with default parameters [29]. Finally, we visualized the collinearity gene pairs of kiwifruit HD-Zip using TBtools [30].

### Cis-elements analysis for kiwifruit HD-Zip genes

To analyze the *cis*-element organization of kiwifruit HD-Zip genes, we obtained the 2000-bp promoter sequences upstream of each HD-Zip gene in kiwifruit using the TBtools software based on the genome sequence and GFF file [37]. We predicted and collected *cis*-elements from the PlantCARE database (http://bioinformatics.psb.ugent.be/webtools/plantcare/html/) [38].

### Expression analysis of kiwifruit HD-Zips

To investigate the expression patterns of HD-Zip genes in different tissues, developmental stages, or stress treatments, we obtained seven published RNA-seq datasets (PRJNA328414, PRJNA514180, PRJNA602928, PRJNA187369, PRJNA691387, PRJNA577204, and PRJNA594489) from the Sequence Read Archive in NCBI (https://www.ncbi.nlm.nih.gov/). We re-analyzed these transcriptome data using the 'Red5' cultivar genomes as reference genome [23, 26]. The reads were aligned using the HISAT2 software (v2.0.1) [39], and the transcripts were assembled and quantified using the STRINGTIE software (v2.1.5) [40].

### Plant material and bacterial strain

The plantlets of *Actinidia chinensis* cultivar ‘Donghong’ were selected for the study and the plant materials were sampled from Guangxi Institute of Botany. The bacterial strain used for the infection experiment was *Pseudomonas syringae* pv. *actinidiae* (Psa) strain C48, isolated from infected kiwifruit plants and characterized for its pathogenicity. Psa inoculation was performed following the protocol previous reported [31].

### RNA extraction and quantitative PCR

RNA was extracted from incubated leaves of each sample at 0 (before injection of bacteria), 2 and 14 DPI following the instructions provided with the HiPure Plant RNA Kits (Magen, Guangzhou, China). RNA quality was monitored on 1% agarose gels. All primers were judiciously designed utilizing the Primer3Plus online software (http://www.bioinformatics.nl/cgi-bin/primer3plus/primer3plus.cgi) and were commercially synthesized by Sangon Biotech Co., Ltd., Shanghai, China (Table S2). The cDNA synthesis from the samples was meticulously conducted through the utilization of the One-step gDNA removal and cDNA synthesis supermix kit (TransGen Biotech Co., Ltd., Beijing, China). Subsequently, this cDNA was employed as the foundational material for all subsequent PCR experiments.

Quantitative PCR (qPCR) assays were executed in a total volume of 20 µL, containing 10 µL of Tip Green qPCR SuperMix (TransGen Biotech Co., Ltd.), 0.2 µM of each primer, 1 µL of cDNA diluted 1:5, and 8.2 µL of ddH2O. The thermal cycling regime consisted of an initial denaturation step at 94 °C for 30 s, followed by 40 amplification cycles at 94 °C for 5 s and 60 °C for 30 s. Subsequently, a gradual temperature increase of 0.5 °C every 10 s was performed to enable melting-curve analysis. Each sample was subjected to triplicate amplification, and all PCR reactions were carried out utilizing the LightCycler 480 instrument (Roche, Basel, Switzerland). The ΔΔCt method was meticulously employed for data analysis, with Achn107181 (kiwifruit Actin gene) serving as the reference gene for normalization.

### Supplementary Information

**Additional file 1:** **Table S1.** Characteristics of kiwifruit HD-Zip genes. **Table S2.** Primers used for RT-PCR and qRT-PCR analysis.

** Additional file 2:** **Figure S1.** Comparison of exon numbers for different HD-Zip genes belonging to different subfamilies. **Figure S2.** Sequence logos for the twelve conserved motifs identified in the kiwifruit HD-Zip gene family. **Figure S3.** Comparison of motif numbers for different HD-Zip genes belonging to different subfamilies. **Figure S4.** The cis-element architectures in the 2000-bp promoter regions of kiwifruit HD-Zips. Rectangles with different colors represented different cis-elements. **Figure S5.** Cis-elements analysis in the promoter regions of kiwifruit HD-Zip genes. The average number of cis-elements for each clade was shown.

## Data Availability

The publicly available RNA sequencing raw data were retrieved at SRA of NCBI with accession PRJNA328414, PRJNA514180, PRJNA602928, PRJNA187369, PRJNA691387, PRJNA577204, and PRJNA594489. All data generated or analyzed during this study are included in this published article and its supplementary information files.
