# Supplementary material for: Genome-wide analysis and expression profiling of the HD-ZIP gene family in kiwifruit
Source: BMC Genomics. 2024 Apr 9;25:354. doi: 10.1186/s12864-024-10025-7 (PMC11003167; doi:10.1186/s12864-024-10025-7)
Supplement: Supplementary file 2 — Additional file 2: Figure S1. Comparison of exon numbers for different HD-Zip genes belonging to different subfamilies. Figure S2. Sequence logos for the twelve conserved motifs identified in the kiwifruit HD-Zip gene family. Figure S3. Comparison of motif numbers for different HD-Zip genes belonging to different subfamilies. Figure S4. The cis-element architectures in the 2000-bp promoter regions of kiwifruit HD-Zips. Rectangles with different colors represented different cis-elements. Figure S5. Cis-elements analysis in the promoter regions of kiwifruit HD-Zip genes. The average number of cis-elements for each clade was shown. [file 12864_2024_10025_MOESM2_ESM.pdf]

Supplementary information

Figure S1 Comparison of exon numbers for different HD-Zip genes belonging to different subfamilies.

Figure S2 Sequence logos for the twelve conserved motifs identified in the kiwifruit HD-Zip gene family.

Figure S3 Comparison of motif numbers for different HD-Zip genes belonging to different subfamilies.

Figure S4 The *cis*-element architectures in the 2000-bp promoter regions of kiwifruit HD-Zips. Rectangles with different colors represented different *cis*-elements.

Figure S5 Cis-elements analysis in the promoter regions of kiwifruit HD-Zip genes. The average number of cis-elements for each clade was shown.

Table S1 Characteristics of kiwifruit HD-Zip genes.

Table S2 Primers used for RT-PCR and qRT-PCR analysis.

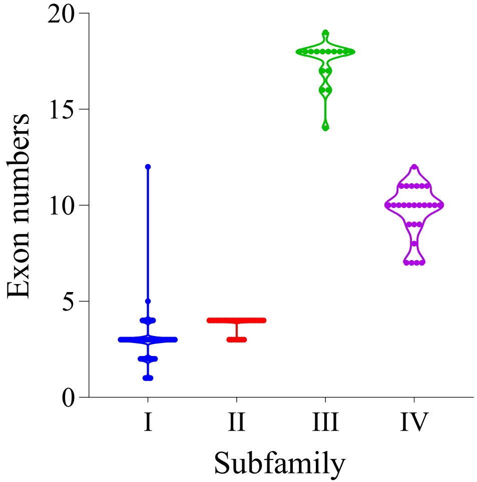

Figure S1 Comparison of exon numbers for different HD-Zip genes belonging to different subfamilies.

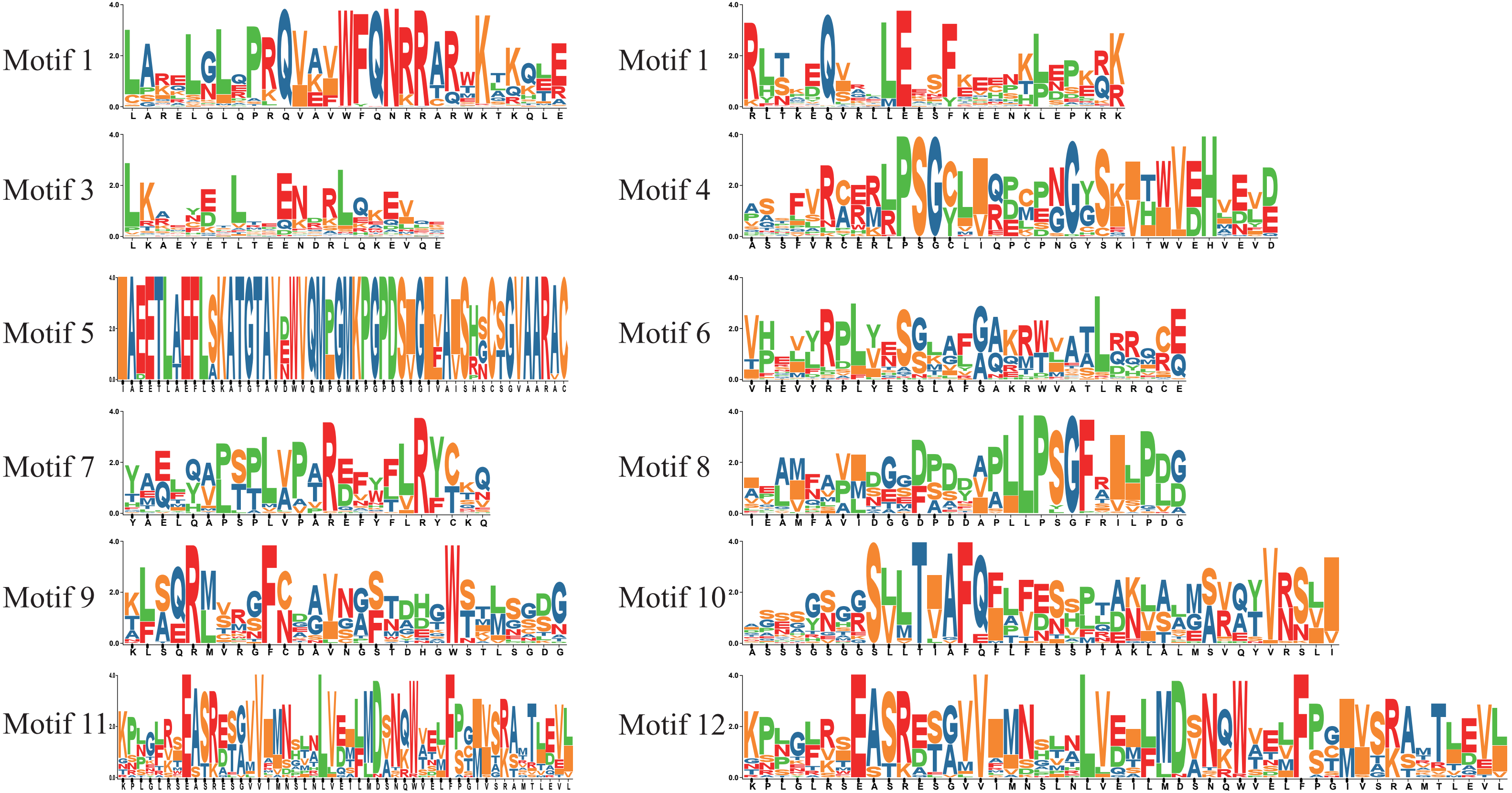

Figure S2 Sequence logos for the twelve conserved motifs identified in the kiwifruit HD-Zip gene family.

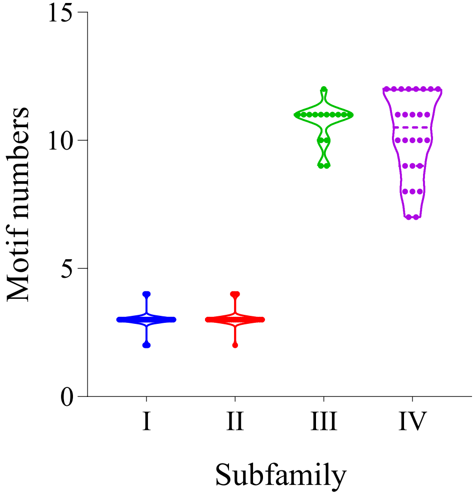

Figure S3 Comparison of motif numbers for different HD-Zip genes belonging to different subfamilies.

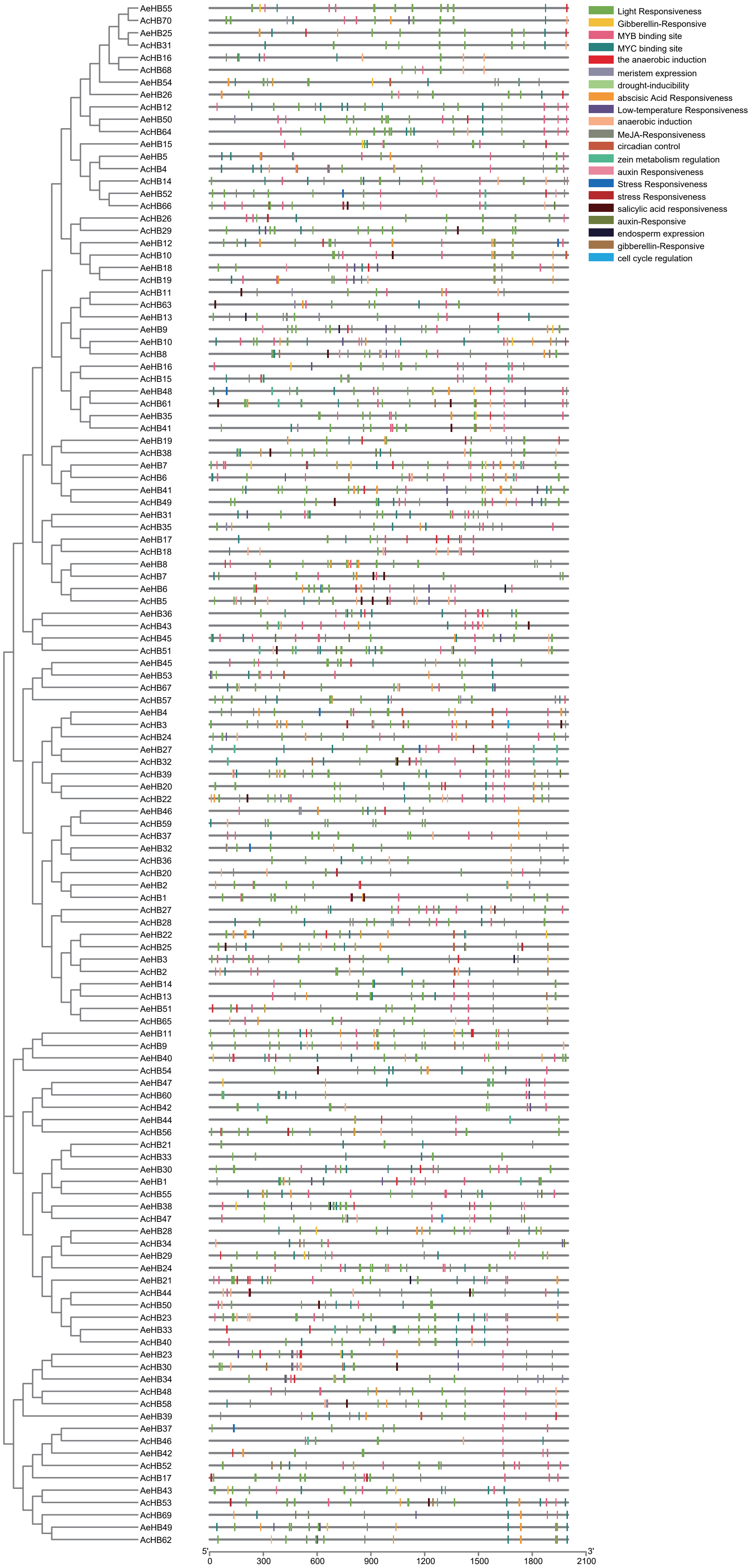

Figure S4 The *cis*-element architectures in the 2000-bp promoter regions of kiwifruit HD-Zips. Rectangles with different colors represented different *cis*-elements.

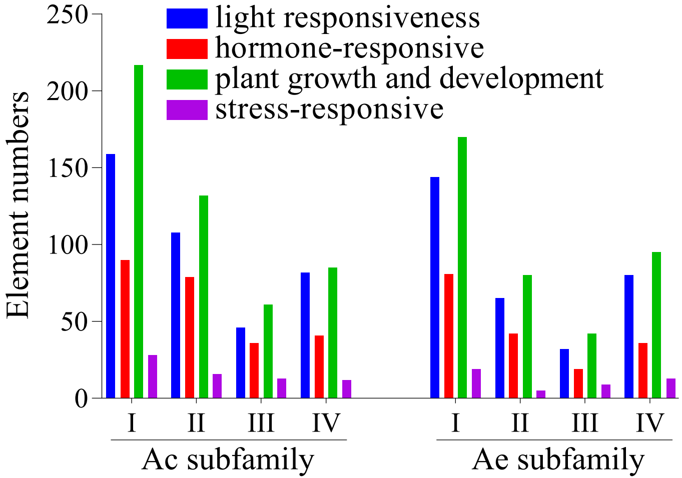

Figure S5 Cis-elements analysis in the promoter regions of kiwifruit HD-Zip genes. The average number of cis-elements for each clade was shown.
